# A new opiine (Hymenoptera, Braconidae) from Australia with discussion of *Diachasma* Foerster

**DOI:** 10.3897/zookeys.437.7726

**Published:** 2014-08-28

**Authors:** Xanthe Shirley, Danielle Restuccia, Andrew Ly, Robert Wharton

**Affiliations:** 1 Department of Entomology, Texas A&M University, College Station, TX 77843

**Keywords:** Lord Howe Island, New Guinea, *Atoreuteus*, *Notiopambolus*

## Abstract

A new species of Opiinae, *Diachasma dentatum* Shirley, Restuccia & Ly, is described from Australia. This species is similar to several other Australian opiines previously described or included in the genus *Diachasma*, but the mandibles are unusually broad, nearly exodont. Notable differences between Australian and Palaearctic *Diachasma* are discussed. *Diachasma tasmaniae* Fischer, 1995, originally described from Tasmania and New South Wales, is newly recorded from Victoria. *Diachasma rufipes* Szépligeti, 1905 is transferred to *Notiopambolus*, new combination.

## Introduction

*Diachasma* Foerster is a morphologically diverse group containing 34 valid, extant species. The geographic range is primarily Holarctic, but the distribution is disjunct, with several species endemic either to eastern Australia or the island of New Guinea. The genus is based on *Diachasma fulgidum* (Haliday), a West Palaearctic species. Basic information on *Diachasma* and its included species can be found in the catalog by Yu et al. (2012). Keys to species are available in Fischer (1972, 1977, 1987, 1988, 1995) and Tobias (1998).

*Diachasma* has traditionally been characterized by the combination of a short second submarginal cell, short clypeus with broadly exposed labrum, occipital carina that is broadly absent dorsal-medially, and relatively complete fore wing venation. *Diachasma* lacks the carapace-like metasoma and clypeal adornments that characterize the few other opiines with these same features. The genus is not demonstrably monophyletic (Tobias 1977, Wharton 1988, 1997) but resolution of this problem will require dense sampling across the subfamily to uncover relationships of the various, seemingly disparate, elements currently residing within *Diachasma*. As is true of all opiines, members of the genus *Diachasma* are parasitoids of cyclorrhaphous Diptera. Three host groups are known, corresponding to three morphologically distinct groups of species. *Diachasma fulgidum* and two other morphologically similar species are parasitoids of leaf-mining flies in the genus *Pegomya* Robineau-Desvoidy (Anthomyiidae) (Fischer 1972, Jimenez et al. 1992), some of which are notable pests. *Diachasma striatum* (Foerster) and *Diachasma wichmanni* (Fischer) have been reared from species of *Chyliza* Fallén (Psilidae) inhabiting twig galls on trees and shrubs (Fischer 1957, Grijpma and van Achterberg 1991). These two species are sometimes placed in *Atoreuteus* Foerster (Wharton 1988, Grijpma and van Achterberg 1991, Quicke et al. 1997), of which *striatum* is the type species. A further three species from the Nearctic Region are parasitoids of fruit-infesting Tephritidae (Muesebeck 1956, Wharton 1997, Wharton and Yoder 2014). One of these, *Diachasma alloeum* (Muesebeck), shows evidence of sequential sympatric speciation in association with its host *Rhagoletis pomonella* (Walsh) (Forbes et al. 2009). Wharton (1997) suggested that these three species may be more closely related to the Nearctic species of *Diachasmimorpha* Viereck that also attack fruit-infesting Tephritidae than to *Diachasma fulgidum*.

The purpose of the present contribution is to describe a new species with unusual mandibular morphology and contrast the New Guinea/Australian fauna with *Diachasma fulgidum* and similar species from the Palaearctic.

## Materials and methods

**Specimens.** Specimens used in this study, including type material of previously described species, were borrowed from or examined at the following institutions: American Entomological Institute, Gainesville, Florida, USA (AEIC), Australian National Insect Collection, Canberra, ACT, Australia (ANIC), Canadian National Collection, Ottawa, Ontario, Canada (CNC), Museum fuer Naturkunde der Humboldt-Universitaet, Berlin, Germany (ZMHB), Hungarian Natural History Museum, Budapest, Hungary (HNHM), Naturhistorisches Museum Wien, Vienna, Austria (NHMW), Texas A&M University Insect Collection, College Station, Texas, USA (TAMU), The Natural History Museum, London, England (BMNH), and U. S. National Museum of Natural History, Washington, D. C., USA (USNM).

In the material examined section, we record label data for the holotype exactly as they appear on the label.

**Figures.** Images were acquired digitally using Syncroscopy’s AutoMontage® software, in combination with a ProgRes 3008 digital camera mounted on a Leica MZ APO dissecting microscope. All images were further processed using various minor adjustment levels in Adobe Photoshop® such as image cropping and rotation, adjustment of contrast and brightness levels, color saturation, and background enhancement. Automontage images are available in color and high resolution at http://mx.speciesfile.org/projects/8/public/site/wharton_lab/home.

**Database management and digital dissemination.** Illustrations and free-text diagnoses for morphospecies were assembled in mx, a web-based content management system that facilitates data management and dissemination for taxonomic and phylogenetic works (e.g. Yoder et al. 2006). The mx project is open source, with code and further documentation available at http://sourceforge.net/projects/mx-database/. Data pertinent to this work, including specimen-level data, images, diagnoses, and descriptions, are available at http://mx.speciesfile.org/projects/8/public/site/wharton_lab/home.

**Terminology, ontology reference, and measurements.** Terminology follows Wharton et al. (2012), wherein terms are linked to the Hymenoptera Anatomy Ontology (HAO, Yoder et al. 2010). Wing cells and abbreviations for wing veins are illustrated in Sharkey and Wharton (1997). Quantitative data in the description are based on all four specimens, unless parts were obscured by positioning on the point mounts. Measurements largely follow Wharton et al. (2012) and Wharton and Norrbom (2013).

## Results and discussion

### Taxonomy

#### Diachasma

Taxon classificationAnimaliaHymenopteraBraconidae

Diachasma Foerster, 1862: 259. Type species: *Opius fulgidus* Haliday, 1837. Monobasic and original designation.
Atoreuteus Foerster, 1862: 241. Type species: *Atoreuteus striatus* Foerster, 1862. Monobasic and original designation. Synonymized by Fischer (1971).
Bathystomus Foerster, 1862: 235. Type species: *Bathystomus xanthopus* Foerster, 1862. Monobasic and original designation. Synonymized by Fischer (1971).
Lytacra Foerster, 1862: 258. Type species: *Lytacra stygia* Foerster, 1862. Monobasic and original designation. Synonymized by Wharton (1987).
Alysopius Tobias, 1976: 76–77. Type species: *Alysopius compressiventris* Tobias, 1976. Monobasic and original designation. Synonymized by Fischer (1986).

##### Diagnosis.

Ventral margin of clypeus even, without teeth or tubercles, truncate to weakly concave; labrum usually flat, always broadly exposed. Occipital carina broadly absent dorsally, present laterally, extending to base of mandible, widely separated from hypostomal carina. Notauli varying from nearly absent to complete and ending in mesoscutal midpit, often sculptured anteriorly; mesoscutal midpit present, varying from punctiform to narrowly elongate. Precoxal sulcus varying from unsculptured and nearly absent to crenulate and extending nearly full length of mesopleuron. Fore wing stigma broad, discrete, with r1 arising from or distad midpoint; second submarginal cell relatively short, with 2RS and 3RSa approximately equal in length; m-cu antefurcal to postfurcal; RS complete to wing margin; 1st subdiscal cell closed (2cu-a present). Hind wing RS poorly developed, often barely indicated distally; m-cu nearly always present, though often weakly developed. First metasomal tergite with or without dorsope. Metasomal tergum 2+3 either smooth or sculptured. Ovipositor sheath varying from barely exserted to extending 0.5 × length of metasoma.

##### Remarks.

Fischer (1971) placed *Atoreuteus* and *Bathystomus* as synonyms of *Diachasma*, but both have been treated separately in subsequent studies (Wharton 1988, Quicke et al. 1997). Belokobylskij et al. (2003) listed them under *Diachasma*, with a note that not all authors agreed with this placement. Yu et al. (2012) also left them in *Diachasma*. Wharton (1988) noted major differences in external morphology between *Atoreuteus* and *Bathystomus*, and these were corroborated by venom gland differences observed by Quicke et al. (1997). Similarly, Wharton (1988) described differences in labral and mandibular morphology that differentiate these two from more typical *Diachasma*. The type species of both *Atoreuteus* and *Bathystomus* also have an exceptionally large pronope. Their placement remains enigmatic, though the shape of the labrum suggests a basal position within Opiinae. They likely merit recognition as separate genera, but we leave them in *Diachasma*, with reluctance, pending a more rigorous analysis of opiine relationships.

The new species described below belongs to an Australian/New Guinea species group that is also morphologically distinct from typical members of *Diachasma* (as represented by species such as *Diachasma fulgidum* and *Diachasma hispanicum* (Fischer)). These austral species include *Diachasma australe* (Fischer), *Diachasma extasis* Fischer, *Diachasma kaltenbachi* Fischer, and *Diachasma tasmaniae* Fischer. This group can be characterized as monophyletic by the distal origin of r1 from the stigma (distinctly beyond the midpoint). The included species can be further recognized by the combination of a postfurcal fore wing m-cu, propodeal sculpture with well-developed median longitudinal carina on anterior half, bordered by relatively unsculptured areas, and T1 with well-developed dorsope. Most species also have fore wing 2CUa shorter than 2cu-a.

The type species, *Diachasma fulgidum*, is unusual in lacking a well-defined malar sulcus and having a relatively flattened scutellum with polished posterior margin.

##### Species excluded.

Specimens of the species newly described below were compared to primary types and other specimens of nominal species of *Diachasma* previously described from Australia and New Guinea. During this examination, we discovered that a specimen in HNHM labeled as the lectotype male of *Diachasma rufipes* Szépligeti, 1905, is a member of the genus *Notiopambolus* van Achterberg & Quicke. The new combination is *Notiopambolus rufipes* (Szépligeti). Several species of *Notiopambolus* have been described from eastern Australia (van Achterberg and Quicke1990, Belokobylskij 1992). It is likely that *rufipes* is a senior synonym of one of these previously described species, but we are unable to place it at this time.

##### Key to species of *Diachasma* known from Australia and New Guinea

**Table:** 

1	Fore wing r1 arising from middle of stigma	2
1'	Fore wing r1 arising distinctly distad middle of stigma	4
2. (1)	Fore wing m-cu postfurcal, entering second submarginal cell	*Diachasma obothorax* Fischer
2'	Fore wing m-cu antefurcal, entering first submarginal cell	3
3. (2’)	Mesosoma 1.33 × longer than high. Temples in dorsal view not strongly receding	*Diachasma gressitti* Fischer
3'	Mesosoma 1.25 × longer than high. Temples in dorsal view strongly receding	*Diachasma anguma* Fischer
4. (1’)	T1 3 × longer than apical width	*Diachasma extasis* Fischer
4'	T1 at most 2 × longer than apical width	5
5. (4’)	Eye in lateral view 5 × longer than temple; mesonotum black	*Diachasma kaltenbachi* Fischer
5'	Eye in lateral view at most 3 × longer than temple; mesonotum predominantly pale: yellow to orange, rarely with one or two weakly infumate spots	6
6. (5’)	Mandible with broad flanges along dorsal and ventral margins basally, abruptly narrowing to apical teeth (Figs 5, 6)	*Diachasma dentatum* Shirley, Restuccia & Ly, sp. n.
6'	Mandible strongly but evenly narrowing to apical teeth, without obvious flanges (Figs 1, 13)	7
7. (6’)	Notauli weakly developed, largely confined to anterior-lateral corners, absent or nearly so on mesoscutal disc; precoxal sulcus unsculptured	*Diachasma australe* (Fischer)
7'	Notauli well developed and deep throughout, extending to midpit posteriorly; precoxal sulcus nearly always crenulate for most or all of its length	*Diachasma tasmaniae* Fischer

**Figures 1–4.**
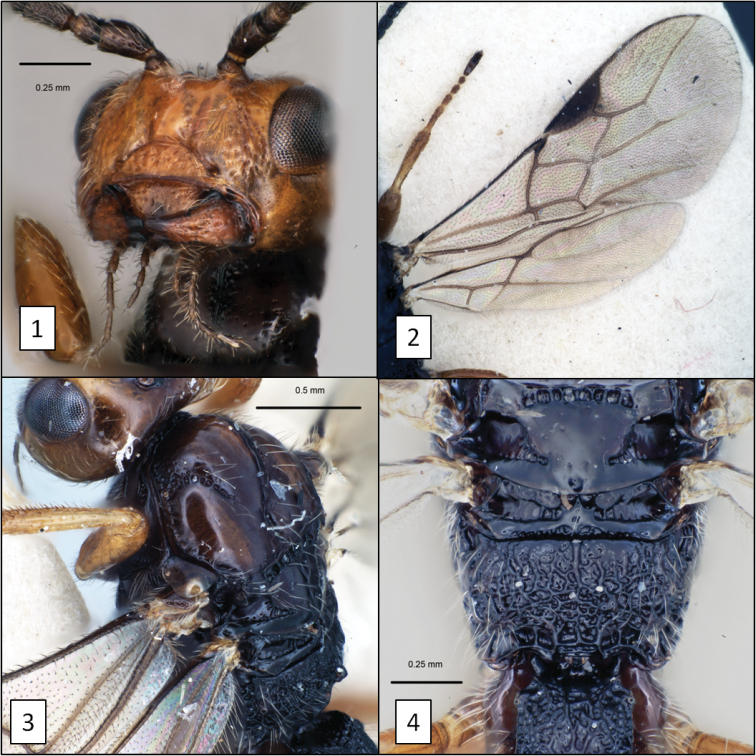
*Diachasma fulgidum* (Haliday) female. **1** Head, frontal view showing exposed labrum and absence of malar sulcus **2** fore and hind wing venation **3** mesosoma, dorsal-lateral view showing notaulus and midpit **4** middle part of body, dorsal view.

**Figures 5–8.**
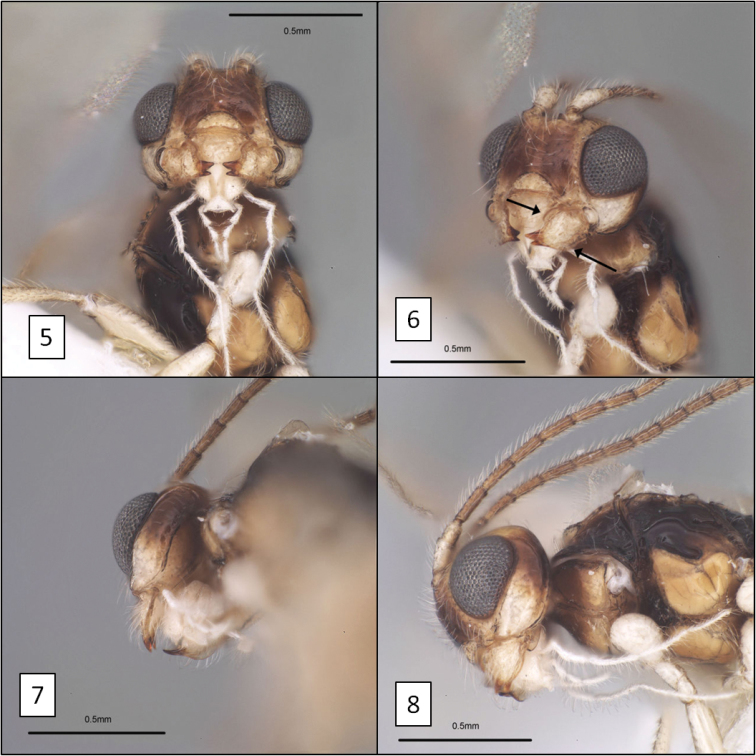
*Diachasma dentatum* Shirley, Restuccia & Ly head, holotype female. **5** Frontal view showing juxtaposition of mandibles **6** mandible, frontal-oblique view, arrows = flanges on dorsal and ventral margins **7** ventral-posterior view showing widely spaced occipital and hypostomal carinae **8** lateral view showing relative size of eye.

**Figures 9–12.**
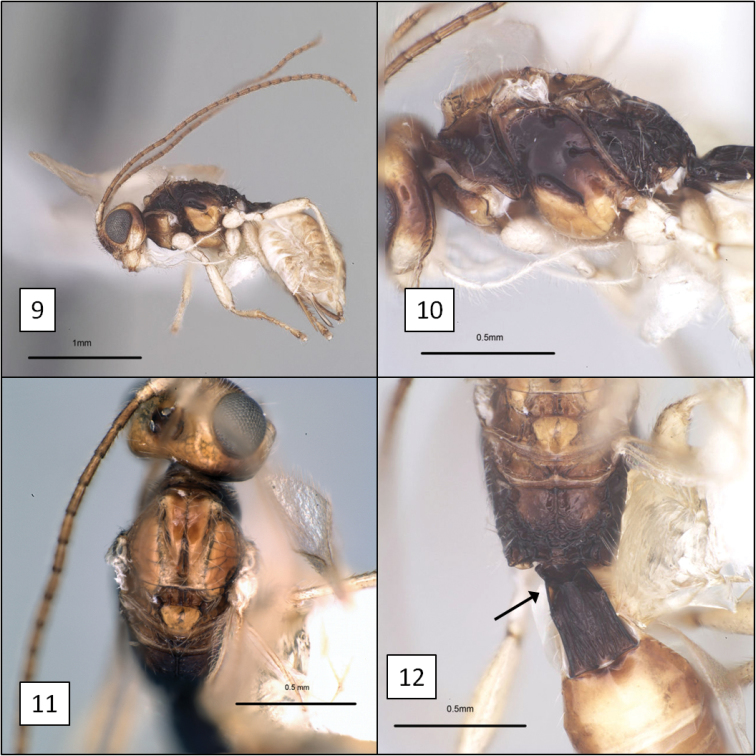
*Diachasma dentatum* Shirley, Restuccia & Ly, holotype female. **9** Lateral habitus **10** Mesosoma, lateral view **11** Mesoscutum, dorsal view **12** Propodeum and T1, dorsal view, arrow = dorsope.

**Figures 13–16.**
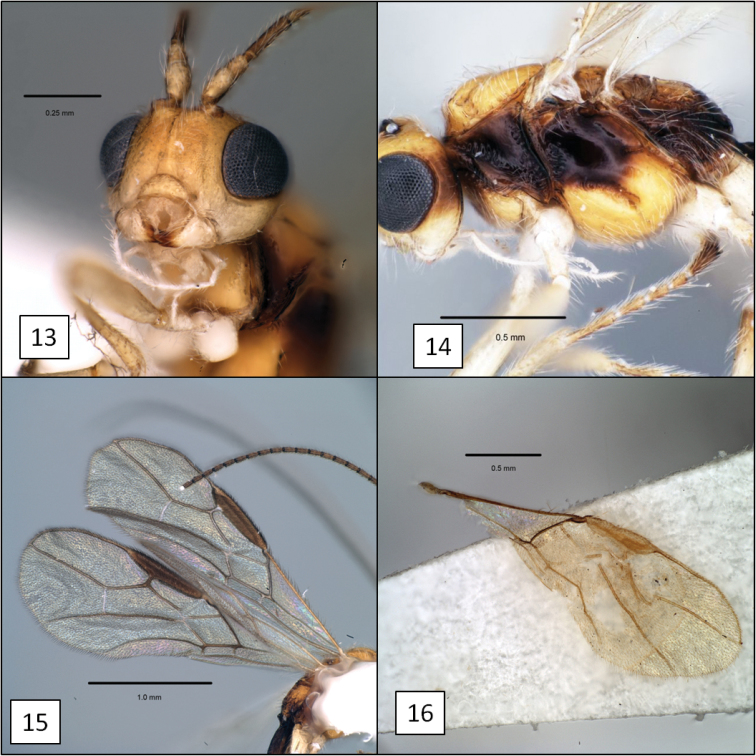
*Diachasma* spp. **13** *Diachasma tasmaniae* Fischer, mandible **14** *Diachasma tasmaniae* mesosoma, lateral view **15** *Diachasma tasmaniae* fore wing **16** *Diachasma dentatum* Shirley, Restuccia & Ly, fore wing stigma, female holotype.

#### Diachasma dentatum

Taxon classificationAnimaliaHymenopteraBraconidae

Shirley, Restuccia & Ly sp. n.

http://zoobank.org/EE48A796-3A5F-49B9-84CC-19587EC17C29

Figs 5 –12 , 16

##### Type locality.

Australia, New South Wales, Lord Howe Island

##### Type material.

Holotype. Female (ANIC), first and only data label, first line: Aust:NSW second line: Lord Howe Is. third line: 17.31.v.1980 fourth line: S.&J.Peck

##### Paratypes.

3 males, same data as holotype (CNC, TAMU).

##### Description.

Face faintly punctate, punctures separated by 5–7 × their diameter, surface weakly shagreened to smooth between punctures. Frons with small, median pit between median ocellus and toruli. Clypeus 2.25–2.65 × wider than high, faintly punctate; ventral margin weakly concave, nearly truncate. Eye moderately large, 2.5–2.75 × longer than temple in lateral view. Malar sulcus distinct, deep throughout; malar space 0.5–0.6 × basal width of mandible. Mandibles deflected ventrally, broadly exposing labrum, apical teeth bent sharply inward relative to base, basal portion as broad as long, with prominent flanges forming dorsal and ventral margins (Figs 5, 6). Antenna with 26 (female) and 30–32 (male) flagellomeres; first flagellomere 3.5–4.0 × longer than wide, 1–1.05 × longer than second. Length of maxillary palps distinctly greater than height of head. Mesosoma 1.5–1.55 × longer than high, 1.85–2.05 × longer than wide. Propleuron smooth, polished, without diagonal sulcus. Pronotum dorsally with small median pit; crenulate groove along posterior margin continuing laterally, extending to ventral margin of pronotum laterally; posterior margin of pronotum laterally crenulate. Notauli deeply impressed, extending from anterior margin to narrowly elongate midpit, crenulate anteriorly, unsculptured on most of disc; supramarginal carina distinct throughout, mesoscutal humeral sulcus crenulate; midpit occupying nearly half length of disc (Fig. 11); median mesoscutal lobe elevated along edge of anterior declivity in holotype. Scuto-scutellar sulcus broad, twice wider than long. Precoxal sulcus (Fig. 10) deep, broad, nearly extending to anterior margin, widely separated from posterior margin, varying from crenulate to almost completely smooth. Propodeum with smooth anterior-lateral areas separated by median longitudinal carina on basal 0.3, carina merging posteriorly with barely discernible median areola largely obscured by fine rugosities covering posterior 0.5 of propodeum. Fore wing stigma discrete, wedge-shaped, gradually widening distally, 4.2–4.5 × longer than wide, r1 arising 0.7 × distance from base, less than half width of stigma at this point; 2RS 1.2 × longer than 3RSa; 3RSb extending to apex of wing; m-cu widely postfurcal; 2CUb arising at or near anterior side of 1st subdiscal cell, 2CUa varying from absent to nearly so. Hind wing RS weak but distinct at base, absent distally; m-cu weakly pigmented and very weakly impressed, long, extending nearly to wing margin, curved basally. Metasoma with T1 1.35–1.45 × longer than apical width, about 1.7 × wider at apex than at base; densely striate, dorsal carinae distinct from base to apex, more nearly parallel-sided and widely separated in male paratypes than female holotype; dorsope large, distinct. T2+3 smooth, very sparsely setose, nearly bare. T2 spiracle on dorsal edge of lateral crease separating median tergite from lateral tergite. Ovipositor sheath about half length of mesosoma, sparsely setose. Color (Figs 9, 10) pale yellow (males) to dark yellow (female) except as follows: pronotum laterally, mesopleuron dorsally, metapleuron, propodeum posteriorly, and T1 dark brown, female more extensively darker than males; ocellar field, most of face, and temple behind eye brown; flagellum and tergal margins light brown; propleuron dorsally and propodeum anteriorly yellow-brown; apical teeth of mandible red; labrum, palps, lower gena, trochanters, and all coxae white, remainder of legs, scape, and pedicel faintly yellow.

##### Diagnosis.

This new species is almost identical morphologically to *Diachasma tasmaniae*, with very similar wing venation, body sculpture, and coloration (including female darker than male). The mandible, however, is distinctly different. The mandible of *Diachasma tasmaniae* is typical of other members of this species group, lacking the flange-like dorsal and ventral margins of the *Diachasma dentatum* mandible. Both *Diachasma tasmaniae* and *Diachasma dentatum* belong to a group of species readily recognized by the combination of a distinct dorsope, r1 arising from the distal portion of the discrete fore wing stigma, and a relatively short second submarginal cell, with 2RS longer than or less commonly equal in length to 3RSa. All other described species in this group have normal mandibles similar to those of *Diachasma tasmaniae* and are darker in color than *Diachasma tasmaniae* and *Diachasma dentatum*.

##### Biology.

Unknown.

##### Etymology.

The species name is Latin for toothed, calling attention to the unusual form of the mandibles.

##### Remarks.

Intraspecific variation in sculpture and quantitative measurements poses challenges for species delineation in this group. The face is more visibly shagreened in two of the males of *Diachasma dentatum*, for example, but the sculpture is weak in all four specimens compared to most but not all specimens of *Diachasma tasmaniae* available for examination (n = 11). The eye varies in size between sexes and among species of this species group, but in known members of this group, the eye is large compared to that of the relatively small eye of *Diachasma fulgidum*.

The placement of *dentatum* in *Diachasma*, along with *tasmaniae* and the other morphologically similar species noted above, will ultimately need to be re-evaluated. There are compelling morphological differences that strongly suggest that these species are not congeneric with the type species, *Diachasma fulgidum*, nor closely resemble the type species of *Atoreuteus* and *Bathystomus*. There are a large number of available genus group names in the Opiinae, including several for species with a well-developed dorsope (as discussed in detail by Wharton 2006) and we are thus reluctant to create a new generic name since one or more of these may prove applicable with a broader analysis of opiine relationships. The described species from Australia and New Guinea are also morphologically diverse, as exemplified by the key to species presented above, and many new species await description, based on material examined in TAMU and ANIC. As noted above, resolution of relationships will necessitate consideration of many opiines currently placed in other genera and is beyond the scope of this work.

Though Lord Howe is an oceanic island well off the coast of New South Wales, this newly described species is remarkably similar in many respects to *Diachasma tasmaniae*. *Diachasma tasmaniae* was originally described from Tasmania and New South Wales (Fischer 1995) and is recorded here for the first time from Victoria (specimens in TAMU and ANIC).

## Supplementary Material

XML Treatment for Diachasma

XML Treatment for Diachasma dentatum
